# Frequency and management of non-cardiac incidental findings on cardiac CT in patients with a suspected stroke

**DOI:** 10.1093/esj/aakaf027

**Published:** 2026-01-03

**Authors:** Chiel F P Beemsterboer, Shan Sui Nio, Berto J Bouma, S Matthijs Boekholdt, Ludo F M Beenen, Henk A Marquering, Charles B L M Majoie, Adrienne van Randen, R Nils Planken, Leon A Rinkel, Jonathan M Coutinho

**Affiliations:** Department of Neurology, Amsterdam UMC, Location University of Amsterdam, Amsterdam, The Netherlands; Department of Neurology, Amsterdam UMC, Location University of Amsterdam, Amsterdam, The Netherlands; Department of Cardiology, Amsterdam UMC, location University of Amsterdam, Amsterdam, The Netherlands; Department of Cardiology, Amsterdam UMC, location University of Amsterdam, Amsterdam, The Netherlands; Department of Radiology and Nuclear Medicine, Amsterdam UMC, location University of Amsterdam, Amsterdam, The Netherlands; Department of Radiology and Nuclear Medicine, Amsterdam UMC, location University of Amsterdam, Amsterdam, The Netherlands; Department of Biomedical Engineering and Physics, Amsterdam UMC, location University of Amsterdam, Amsterdam, The Netherlands; Department of Radiology and Nuclear Medicine, Amsterdam UMC, location University of Amsterdam, Amsterdam, The Netherlands; Department of Radiology and Nuclear Medicine, Amsterdam UMC, location University of Amsterdam, Amsterdam, The Netherlands; Department of Radiology and Nuclear Medicine, Mayo Clinic, Rochester, Minnesota, United States; Department of Neurology, Amsterdam UMC, Location University of Amsterdam, Amsterdam, The Netherlands; Department of Neurology, Amsterdam UMC, Location University of Amsterdam, Amsterdam, The Netherlands

**Keywords:** incidental findings, acute ischaemic stroke, suspected stroke, cardiac computed tomography

## Abstract

**Introduction:**

Cardiac CT is increasingly used to screen for cardioembolism in stroke patients. We assessed the frequency and management of non-cardiac incidental findings on prospective ECG-gated cardiac CT in patients with a suspected stroke.

**Patients and methods:**

This was a post-hoc analysis of the Mind the Heart study, a prospective single-centre cohort study including consecutive adult patients with acute ischaemic stroke (AIS), transient ischaemic attack (TIA), or a stroke mimic who underwent cardiac CT as part of an acute stroke imaging protocol. Endpoints were pre-defined non-cardiac incidental findings that were detected on cardiac CT: pulmonary embolism (PE), potential malignant lesions, pulmonary consolidations or ground-glass densities, bone fractures, lymphadenopathy, focal liver lesions, and ascending aortic or pulmonary artery dilatation. Change of management was defined as additional treatment or follow-up.

**Results:**

We included 654 patients (57% men, median age 71 [IQR 59–80] years) of whom 451 (69%) had AIS, 48 had TIA (7%), and 155 had a stroke mimic (24%). Overall, 58 non-cardiac incidental findings were found in 55 (8%; 95%CI, 6–11) patients. The most frequent incidental findings were consolidations or ground-glass densities (*n* = 17, 3%), liver cysts or non-specific hypodensities (*n* = 14, 2%), pulmonary nodules or masses (*n* = 9, 1%), and PEs (*n* = 8, 1%). Incidental findings led to a change of management in 17/55 (31%) patients of whom 13/55 (24%) had additional follow-up and 9/55 (16%) received treatment (anticoagulation *n* = 8, chemotherapy *n* = 1).

**Discussion & Conclusion:**

Non-cardiac incidental findings were observed on cardiac CT in 8% of patients with a suspected stroke. These findings changed management in 31% of these patients.

## Graphical Abstract

**Graphical Abstract:**
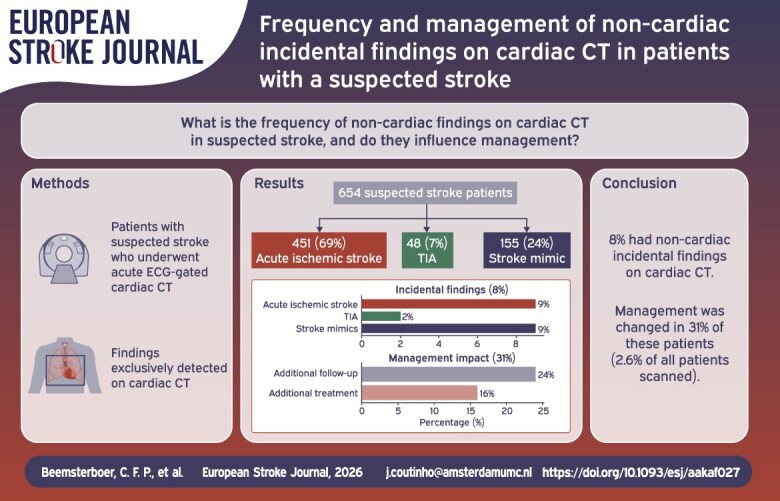

## Introduction

Cardiac CT is increasingly used to screen for cardioembolism in patients with acute ischaemic stroke (AIS).^1^^,^^2^ Previous studies have shown that it is technically feasible to implement cardiac CT as part of an initial stroke imaging protocol (acute cardiac CT),^3–8^ and that acute cardiac CT has a higher diagnostic yield compared to transthoracic echocardiography for the detection of high-risk structural sources of embolism.^1^ Besides the additional diagnostic value of cardiac CT to detect cardiac sources of embolism, the extended field of view may also result in the detection of thoracic and abdominal pathology.^9^^,^^10^ While this can lead to the identification of clinically relevant pathology, incidental findings may also lead to overtreatment, unnecessary follow-up, increased healthcare costs, and patient anxiety.^11^^,^^12^

While some incidental findings may result in high-value care and improved patient outcomes, the majority lack clinical relevance, and determining their significance at the time of detection remains difficult.^11^ For this study, we aimed to assess the frequency and management of non-cardiac incidental findings detected on cardiac CT in patients with a suspected stroke.

## Patients and methods

### Study design and patient population

This was a post-hoc analysis of the Mind the Heart study, a prospective single-centre cohort study on the diagnostic yield of cardiac CT in patients with AIS. The design and main results of this study have been previously published.^1^ Briefly, between May 2018 and November 2020, consecutive adult patients who presented to the emergency department with suspected AIS who underwent cardiac CT as part of the acute imaging protocol.^13^ The medical ethics committee of Amsterdam UMC, approved the study (2018_017#C2018275 and W21_027# 21.031). All patients or their legal representatives provided written informed consent.

### Diagnostic procedures

For image acquisition, a third-generation dual-source CT scanner (Somatom Force, Siemens Healthineers, Erlangen, Germany) situated at the emergency department was used. After clinical examination, patients sequentially underwent non-contrast CT of the brain, CT perfusion, and non-gated CT-angiography of the aortic arch, cervical, and intracranial arteries. Immediately thereafter, prospective ECG-gated sequential cardiac CT was performed in end-diastole after repositioning the patient’s arms above the head. The on-call radiologist reported the CT scans of the brain and extracranial and intracranial arteries and screened for non-cardiac incidental findings on cardiac CT. Subsequently, a predefined comprehensive assessment of the cardiac CT, including screening for incidental non-cardiac findings, was performed by a cardiac radiologist (R.N.P. or A.v.R.) for research purposes.

### Data collection

Incidental findings are defined as unexpected abnormalities detected on imaging and unrelated to the patient’s symptoms. Endpoints were the following non-cardiac incidental findings that were exclusively detected on cardiac CT (ie, not visible on any of the other images): pulmonary embolism (PE), potential malignant thoracic or abdominal lesions, pulmonary consolidations or ground-glass densities, bone fractures, lymphadenopathy, focal liver lesions, and ascending aortic or pulmonary artery dilatation. Findings such as emphysema, atelectasis, pleural fluid, and diaphragmatic hernia were not scored as incidental finding. For the following findings, size cut-offs were used to determine the necessity for additional diagnostic work up: pulmonary nodules ≥6 mm were defined as significant based on the Fleischner criteria^14^; mediastinal lymphadenopathy was defined as lymph node with a short axis ≥10 mm.^15^ Medical records were reviewed retrospectively to assess any changes in management resulting from these findings, defined as additional diagnostic procedures, follow-up and treatment. Moreover, findings identified on previous imaging were excluded.

### Statistical analysis

We compared baseline characteristics and the proportion of incidental findings between patients with AIS, TIA, and stroke mimics. We compared continuous variables with a Kruskal–Wallis test, and categorical variables with a χ^2^ test or a Fisher’s exact test as appropriate. Analyses were performed using R, version 4.4.2 (R foundation for Statistical Computing, 2023). For all tests, we used a 2-sided significance level of 0.05.

## Results

**Figure 1:**
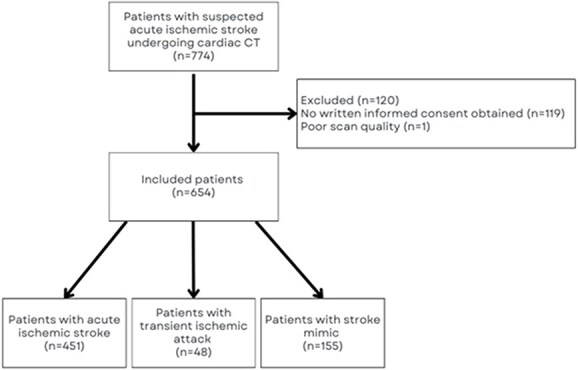
Flowchart of patients.

Between May 2018 and November 2020, 774 patients with a suspected acute ischaemic stroke underwent a cardiac CT as part of the initial stroke imaging protocol. Of these, 119 patients were excluded because no informed consent could be obtained and one patient was excluded because of poor scan quality of the cardiac CT (Figure 1). Therefore, we included 654 patients (43% female, median age 71 years [IQR 59–80]), of whom 451 (69%) had AIS, 155 a stroke mimic (24%) and 48 a TIA (7%) (Table 1). Baseline characteristics stratified according to final diagnosis are presented in Table S1.

**Table 1:** Baseline characteristics

	**Study population** **(*n* = 654)**
Age, median (IQR)	71 (59-80)
Sex (male), *n* (%)	372 (57)
Medical history, *n* (%)	
Ischaemic stroke	130 (19.9)
TIA	66 (10.1)
Atrial fibrillation	112 (17.1)
Diabetes mellitus	102 (15.6)
Hypertension	303 (46.3)
Hypercholesterolemia	101 (15.4)
Myocardial infarction	78 (11.9)
Malignancy	100 (15.3)
Index diagnosis	
Acute ischaemic stroke, *n* (%)	451 (69)
TIA, *n* (%)	48 (7)
Stroke mimic, *n* (%)	155 (24)
Seizure	45 (29)
Peripheral vestibulopathy	22 (14)
Functional neurological symptoms	16 (10)
Syncope or cardiac cause	10 (6)
Intoxication/metabolic	10 (6)
Other^b^	46 (30)
Unknown	6 (4)
Reperfusion therapy, *n* (%)^a^	
Intravenous thrombolysis	184 (40.8)
Endovascular thrombectomy	101 (22.4)
High-risk cardioembolic source on cardiac CT	58 (8.9)

Abbreviations: IQR: interquartile range, TIA: transient ischaemic stroke

a Reperfusion therapy in acute ischaemic stroke group.

b Other includes infection (*n* = 9), benign headache (*n* = 8), hypertension (*n* = 5), musculoskeletal symptoms (*n* = 4), tumour/metastasis (*n* = 4), hyperventilation syndrome (*n* = 2), delirium (*n* = 1), dementia (*n* = 1), other (*n* = 12).

**Figure 2:**
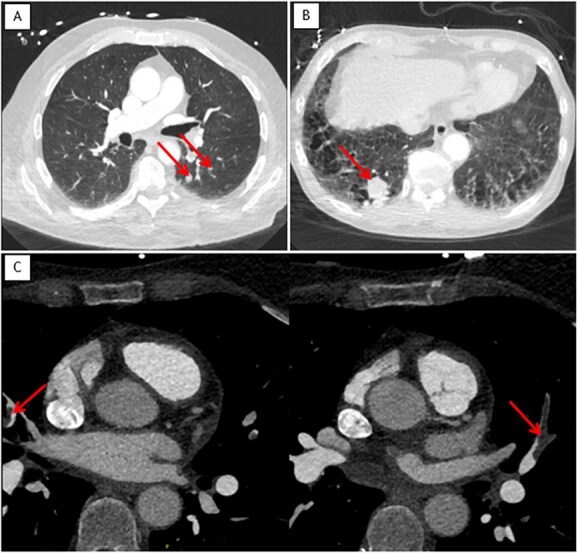
Example cases of non-cardiac incidental findings on cardiac-CT**.** Cardiac CT showing (arrows). (A) Two pulmonary nodules suspicious for malignancy of 11 and 7 mm in the left lower lobe. (B) A pulmonary mass of 25 mm suspicious for malignancy in the right lower lobe. (C) Bilateral segmental pulmonary embolism.

**Figure 3:**
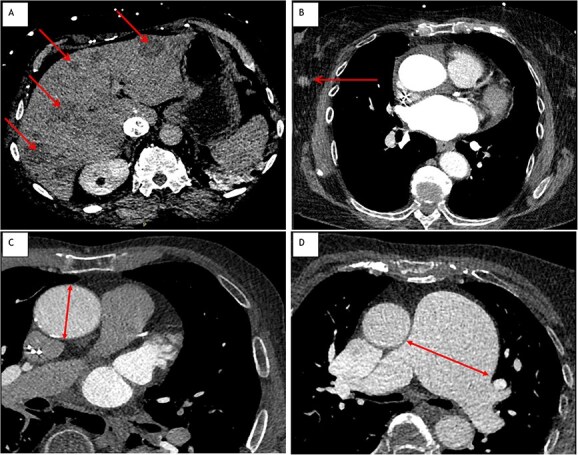
Example cases of non-cardiac incidental findings on cardiac-CT**.** Cardiac CT showing (arrows). (A) Multiple liver hypodensities suspicious for metastatic disease. (B) A mass suspicious for malignancy in the right breast. (C) Dilatated ascending aorta of 46 mm. (D) Severely dilatated pulmonary truncus of 64 mm probably as result of pulmonary hypertension caused by partial anomalous pulmonary venous return.

Overall, 58 non-cardiac incidental findings were observed in 55 (8%; 95% CI, 6–11) patients. Incidental findings were: PEs (*n* = 8), consolidations (*n* = 8), ground-glass densities (*n* = 9), pulmonary nodules (*n* = 7), pulmonary masses (*n* = 2), lymphadenopathy (*n* = 3), dilatated ascending aorta (*n* = 1), dilatated pulmonary artery (*n* = 2), liver mass (*n* = 1), liver cyst or non-specific hypodensity (*n* = 14), breast lesion (*n* = 1), and osseous fracture (*n* = 1) (Table 2). Incidental findings were detected in 9% of patients with AIS, in 2% of patients with TIA and 9% of patients with stroke mimics (*P* = .26). Examples of incidental findings are presented in Figures 2 and 3.

**Table 2:** Non-cardiac incidental findings

	**Total population (*n* = 654)**
Total patients with a finding, *n* (%)	55 (8.4)
Total findings, *n* (%)	58 (8.9)
Lungs, *n* (%)	34 (5.2)
Pulmonary embolism	8 (1.2)
Consolidation	8 (1.2)
Ground glass density	9 (1.4)
Nodule	7 (1.1)
Mass	2 (0.3)
Liver, *n* (%)	15 (2.3)
Nodule	0 (0)
Mass	1 (0)
Cyst	13 (2.0)
Non-specific hypodensity	1 (0.2)
Osseous, *n* (%)	2 (0.3)
Lesion suspect for malignancy	0 (0)
Fracture	1 (0.2)
Non-specific sclerotic lesion	1 (0.2)
Lymph nodes, *n* (%)	3 (0.5)
Breast lesions suspect for malignancy, *n* (%)	1 (0.2)
Ascending aortic aneurysm, *n* (%)	1 (0.2)
Dilatated pulmonary artery, *n* (%)	2 (0.3)

In 17 of 55 patients (31%), incidental findings led to a change of management. In 13 of 55 (24%) patients, additional diagnostic work-up or follow-up was performed and in 9 of 55 (16%), the incidental finding necessitated treatment (anticoagulation *n* = 8, chemotherapy *n* = 1).

The finding which most frequently influenced management was the detection of a PE. This was detected in 8 of 654 (1.2%) patients and led to initiation of anticoagulation in 7 of 8 cases. One patient died before anticoagulation could be initiated. No bleeding complications were observed in patients with PE in whom anticoagulation therapy was initiated. In total, 4 of 8 patients with a PE had a known active malignancy at the time of presentation. Of the 8 patients with PE, 3 patients had a possible patent foramen ovale (PFO) detected on acute cardiac CT. However, none of these patients underwent transthoracic echocardiography (TTE) with agitated saline contrast to confirm these findings.

Pulmonary findings influenced management in 3 of 8 patients with pulmonary consolidations (antibiotic treatment *n* = 2, additional CT *n* = 1). One patient with pulmonary nodules underwent follow-up imaging that ruled out a malignancy. In 1 patient who had died and presented with 2 suspicious pulmonary nodules, autopsy revealed pulmonary metastases. In another patient, cardiac CT suggested primary lung carcinoma with liver metastasis, leading to chemotherapy initiation (Table 3).

**Table 3:** Change of management due to non-cardiac incidental findings

**Findings**	**Findings,** ***n* (%)**	**Change of management, *n* (%)**	**Treatment, *n* (%)**	**Diagnostics, *n* (%)**
All findings	58	17 (29)	9 (16)	13 (22)	
Lungs					
Total	34 (59)	13 (39)	8 (24)	10 (29)	
Embolism	8 (14)	7 (88)	7 (88)	5 (63)	
Consolidation	8 (14)	3 (43)	2 (25)	2 (25)	
Ground glass density	9 (16)	0 (0)	0 (0)	0 (0)	
Nodule	7 (12)	1 (14)	0 (0)	1 (0)	
Mass	2 (4)	2 (100)	1 (50)	2 (100)	
Liver					
Total	15 (26)	2 (13)	1 (7)	1 (7)	
Nodule	0 (0)	0 (0)	0 (0)	0 (0)	
Mass	1 (2)	1 (100)	1 (100)	0 (0)	
Cyst	13 (22)	1 (8)	0 (0)	1 (8)	
Non-specific hypodensity	1 (2)	0 (0)	0 (0)	0 (0)	
Osseous					
Total	2 (4)	0 (0)	0 (0)	0 (0)	
Lesion suspect for malignancy	0 (0)	0 (0)	0 (0)	0 (0)	
Fracture	1 (2)	0 (0)	0 (0)	0 (0)	
Non-specific sclerotic lesion	1 (2)	0 (0)	0 (0)	0 (0)	
Lymph Nodes	3 (5)	1 (33)	0 (0)	1 (33)	
Breast lesion suspect for malignancy	1 (2)	1 (100)	0 (0)	1 (100)	
Ascending aortic dilatation/aneurysm	1 (2)	0 (0)	0 (0)	0 (0)	
Dilatated pulmonary artery	2 (3)	0 (0)	0 (0)	0 (0)	

## Discussion

In a cohort of patients with suspected AIS who underwent cardiac CT as part of the routine acute imaging protocol, a non-cardiac incidental finding was detected in 1 of 13 patients. Detection rate did not differ significantly between patients with AIS, TIA, or a stroke mimic, although numerically the rate was lower in patients with TIA, which may be explained by limited statistical power in this group. In approximately one-third of patients, detection of the incidental finding led to a change in patient management, either additional investigations or targeted treatment. The finding that most often resulted in a change of clinical management was a PE, which was detected in approximately 1% of patients.

In a similar study among 1111 patients with suspected stroke, an extra-cardiac incidental finding was found in 15.6% of patients on cardiac CT, approximately twice as often as in our study. The higher prevalence in their study can be explained by the fact that the most commonly observed findings, pulmonary artery dilatation and aortic aneurysms (detected in 10.2% and 4.6%, respectively), are usually also identified on standard CT angiography of the cervical arteries rather than exclusively on cardiac CT, and thus were excluded in our study. The authors reported a change in management in 11.5% (20 of 173) patients with an incidental finding. Pulmonary and breast lesions and PE most often led to changes in management, while pulmonary artery dilatation and aortic aneurysms rarely did. As opposed to our study, incidental findings were also more often observed in patients with AIS compared to non-stroke patients, but this difference resulted from differences in prevalence of pulmonary artery dilatation and aortic aneurysms.^9^

Lee et al. reported higher rates of PE (7.9%) and malignant findings (6.7%) in 89 acute ischaemic stroke patients undergoing non-ECG gated cardiac CT, with PE attributed to a PFO in 3 cases.^10^ The authors attributed the relatively high prevalence of these findings to non-consecutive enrolment and small sample size (*n* = 89).

In our study, no PFO detected on cardiac CT in patients with a PE was confirmed by TTE with saline agitated contrast. Our previous studies showed that prospective ECG-gated cardiac CT is not a suitable screening method due to a low sensitivity, thereby, we found that some of the cases with a possible PFO on cardiac CT had a negative TTE (positive predictive value 59% [95% CI, 14–95]).^16^

Incorporating cardiac CT in the diagnostic work-up of acute ischaemic stroke leads to detection of similar proportions of high risk cardioembolic sources (8.9%) and incidental findings (8.4%).^1^ While some of these incidental findings are clearly clinically relevant, it remains important to consider the potential implications of overdiagnosis of incidental findings. Incidental findings can also lead to unnecessary follow-up procedures, increased healthcare costs, and additional patient distress without clear clinical relevance.^11^^,^^12^ Future studies should investigate the trade-off between detecting clinically relevant and irrelevant incidental findings.

While ECG-gated cardiac CT provides superior image quality by reducing motion artefacts, incidental extracardiac findings such as pulmonary nodules or emboli, and liver lesions are also frequently detected on non-ECG-gated cardiac CT scans. Previous studies have reported a prevalence of such findings of up to 30%.^17^ However, no prior research has specifically investigated non-cardiac incidental findings on non-ECG-gated cardiac CT in patients with suspected stroke. Therefore, the prevalence of non-cardiac incidental findings observed in this study is likely generalizable to other non-gated CT protocols.

A strength of this study is that we exclusively described findings detected through cardiac CT, thereby providing an accurate assessment of the additional diagnostic value of detection of extracardiac findings by incorporating cardiac CT into the diagnostic work-up of patients with suspected stroke. This study also has several limitations. First, this is a single-centre study with a limited sample size which limits the generalizability of our findings and also limits our ability to draw conclusions about rare incidental findings on cardiac CT. Second, as this was a post-hoc analysis of an observational study, the true impact of the findings on management is difficult to ascertain. In our retrospective analysis of clinical management there was no loss to follow-up or missing outcome data. Nevertheless, the retrospective design limits assessment of long-term clinical impact, and future studies are warranted to evaluate the relevance of these findings over time. Third, while we selected non-cardiac findings that could potentially lead to a change in management based on findings reported in other studies, this selection remains somewhat subjective, which could lead to over- or underestimation of the proportion of clinically relevant findings. Coronary artery disease was not systematically assessed in current study. Future research including systematic evaluation of the coronary arteries could provide valuable insights into the clinical significance of cardiac incidental findings.

## Conclusion

In 8% of patients with a suspected stroke, cardiac CT identified a non-cardiac incidental finding. These findings changed management in around one-third of these patients. This provides important information on the frequency and implications of extra-cardiac findings when implementing cardiac CT as part of the acute stroke work-up.

## Supplementary Material

Supplemental_material_Incidental_findings_Mind_the_Heart_aakaf027

## Data Availability

The data that support the findings of this study are available from the corresponding author upon reasonable request.
