# Supplementary material for: Frequency and management of non-cardiac incidental findings on cardiac CT in patients with a suspected stroke
Source: Eur Stroke J. 2026 Jan 3;11(1):aakaf027. doi: 10.1093/esj/aakaf027 (PMC12866668; doi:10.1093/esj/aakaf027)
Supplement: Supplemental_material_Incidental_findings_Mind_the_Heart_aakaf027 [file supplemental_material_incidental_findings_mind_the_heart_aakaf027.docx]

Supplemental material

**Frequency and management of non-cardiac incidental findings on cardiac CT in patients with a suspected stroke**

Chiel F.P. Beemsterboer, MD,^a^ Shan Sui Nio, MD,^a^ Berto J. Bouma, MD, PhD, ^b^ S. Matthijs Boekholdt, MD, PhD,^b^ Ludo F.M. Beenen, MD, PhD,^b^ Henk A. Marquering, PhD,^b,d^ Charles B.L.M. Majoie, MD, PhD,^b^ Adrienne van Randen, MD, PhD,^b^ R. Nils Planken, MD, PhD,^e^ Leon A. Rinkel, MD,^a*^ Jonathan M. Coutinho, MD, PhD^a *^

**Contents Page**

**Table S1: Baseline characteristics, difference between subgroups** Page 2

**Table S2: Non-cardiac incidental findings, difference between subgroups** Page 3

Table S1: Baseline characteristics, difference between subgroups

|  | Total population  n=654 | AIS*  n=451 | TIA  n=48 | SMi  n=155 |
| --- | --- | --- | --- | --- |
| Age (median [IQR]) | 71 [59-80] | 72 [62-81] | 73 [63-80] | 70 [59- 80] |
| Sex (male), n (%) | 372 (57) | 268 (59) | 33 (69) | 71 (56) |
| Medical history, n (%) |  |  |  |  |
| Ischemic stroke | 130 (19.9) | 82 (18.2) | 10 (20.8) | 38 (24.5) |
| TIA | 66 (10.1) | 41 (9.1) | 11 (22.9) | 14 (9.0) |
| Atrial fibrillation | 112 (17.1) | 77 (17.1) | 5 (10.4) | 30 (19.4) |
| Diabetes Mellitus | 102 (15.6) | 72 (16.0) | 6 (12.5) | 24 (15.5) |
| Hypertension | 303 (46.3) | 209 (46.3) | 31 (64.6) | 63 (40.6) |
| Hypercholesterolemia | 101 (15.4) | 70 (15.6) | 13 (27.1) | 18 (11.6) |
| Myocardial infarction | 78 (11.9) | 59 (13.1) | 9 (18.8) | 10 (6.5) |
| Malignancy | 100 (15.3) | 65 (14.5) | 6 (12.5) | 29 (18.7) |
| High risk cardioembolic source on cardiac CT, n (%) | 58 (8.9) | 52 (11.5) | 4 (8.3) | 2 (1.3) |

AIS = acute ischemic stroke, TIA = transient ischemic attack, SMi = stroke mimics

*In the AIS group, 7 patients had more than one high-risk cardioembolic source

Table S2: Non-cardiac incidental findings, difference between subgroups

|  | Total population (n=654) | AIS  (n=451) | TIA  (n=48) | SMi  (n=155) |
| --- | --- | --- | --- | --- |
| Total findings, n (%) | 58 (8.9) | 40 (8.9) | 1 (2.1) | 14 (9.0) |
| Lungs, n (%) | 34 (5.2) | 26 (5.8) | 0 (0) | 8 (5.2) |
| Pulmonary embolism | 8 (1.2) | 7 (1.6) | 0 (0) | 1 (0.6) |
| Consolidation | 8 (1.2) | 5 (1.1) | 0 (0) | 3 (1.9) |
| Ground glass density | 9 (1.4) | 9 (2.0) | 0 (0) | 0 (0) |
| Nodule | 7 (1.1) | 5 (1.1) | 0 (0) | 2 (1.3) |
| Mass | 2 (0.3) | 0 (0) | 0 (0) | 2 (1.3) |
| Liver, n (%) | 15 (2.3) | 9 (2.0) | 1 (2.1) | 5 (3.2) |
| Nodule | 0 (0) | 0 (0) | 0 (0) | 0 (0) |
| Mass | 1 (0) | 0 (0) | 0 (0) | 1 (0.6) |
| Cyst | 13 (2.0) | 8 (1.8) | 1 (2.1) | 4 (2.6) |
| Nonspecific hypodensity | 1 (0.2) | 1 (0.2) | 0 (0) | 0 (0) |
| Osseous, n (%) | 2 (0.3) | 2 0.4) | 0 (0) | 0 (0) |
| Lesion suspect for malignancy | 0 (0) | 0 (0) | 0 (0) | 0 (0) |
| Fracture | 1 (0.2) | 1 (0.2) | 0 (0) | 0 (0) |
| Non-specific sclerotic lesion | 1 (0.2) | 1 (0.2) | 0 (0) | 0 (0) |
| Lymph nodes, n (%) | 3 (0.5) | 1 (0.2) | 0 (0) | 2 (1.3) |
| Breast lesions suspect for malignancy, n (%) | 1 (0.2) | 0 (0) | 0 (0) | 1 (0.6) |
| Ascending aortic aneurysm, n (%) | 1 (0.2) | 1 (0.2) | 0 (0) | 0 (0) |
| Dilatated pulmonary artery, n (%) | 2 (0.3) | 2 (0.4) | 0 (0) | 0 (0) |

AIS = acute ischemic stroke, TIA = transient ischemic attack, SMi = stroke mimics
